# Dr. Francis Waters Gallagher

**Published:** 1915-10

**Authors:** 


**Dr. Francis Waters Gallagher,** Buffalo 1877, died at his home in Los Angeles, July 20, aged 62. Until two years ago, he practiced in El Paso.

